# Activation of WNT / β-Catenin Signaling in Pulmonary Fibroblasts by TGF-β_1_ Is Increased in Chronic Obstructive Pulmonary Disease

**DOI:** 10.1371/journal.pone.0025450

**Published:** 2011-09-30

**Authors:** Hoeke A. Baarsma, Anita I. R. Spanjer, Gertruud Haitsma, Lilian H. J. M. Engelbertink, Herman Meurs, Marnix R. Jonker, Wim Timens, Dirkje S. Postma, Huib A. M. Kerstjens, Reinoud Gosens

**Affiliations:** 1 Department of Molecular Pharmacology, University of Groningen, Groningen, The Netherlands; 2 Department of Pathology, University Medical Center Groningen, University of Groningen, Groningen, The Netherlands; 3 Department of Pulmonology, University Medical Center Groningen, University of Groningen, Groningen, The Netherlands; Helmholtz Zentrum München/Ludwig-Maximilians-University Munich, Germany

## Abstract

**Background:**

Chronic obstructive pulmonary disease (COPD) is characterized by abnormal extracellular matrix (ECM) turnover. Recently, activation of the WNT/β-catenin pathway has been associated with abnormal ECM turnover in various chronic diseases. We determined WNT-pathway gene expression in pulmonary fibroblasts of individuals with and without COPD and disentangled the role of β-catenin in fibroblast phenotype and function.

**Methods:**

We assessed the expression of WNT-pathway genes and the functional role of β-catenin, using MRC-5 human lung fibroblasts and primary pulmonary fibroblasts of individuals with and without COPD.

**Results:**

Pulmonary fibroblasts expressed mRNA of genes required for WNT signaling. Stimulation of fibroblasts with TGF-β_1_, a growth factor important in COPD pathogenesis, induced WNT-5B, FZD_8_, DVL3 and β-catenin mRNA expression. The induction of WNT-5B, FZD_6_, FZD_8_ and DVL3 mRNA by TGF-β_1_ was higher in fibroblasts of individuals with COPD than without COPD, whilst basal expression was similar. Accordingly, TGF-β_1_ activated β-catenin signaling, as shown by an increase in transcriptionally active and total β-catenin protein expression. Furthermore, TGF-β_1_ induced the expression of collagen1α1, α-sm-actin and fibronectin, which was attenuated by β-catenin specific siRNA and by pharmacological inhibition of β-catenin, whereas the TGF-β_1_-induced expression of PAI-1 was not affected. The induction of transcriptionally active β-catenin and subsequent fibronectin deposition induced by TGF-β_1_ were enhanced in pulmonary fibroblasts from individuals with COPD.

**Conclusions:**

β-catenin signaling contributes to ECM production by pulmonary fibroblasts and contributes to myofibroblasts differentiation. WNT/β-catenin pathway expression and activation by TGF-β_1_ is enhanced in pulmonary fibroblasts from individuals with COPD. This suggests an important role of the WNT/β-catenin pathway in regulating fibroblast phenotype and function in COPD.

## Introduction

Chronic obstructive pulmonary disease (COPD) is characterized by progressive airflow limitation, which is associated with an abnormal inflammatory response of the lungs to noxious particles or gases. Long-term exposure to cigarette smoke is the major risk factor for the development of COPD [1], [2]. Progressive loss of lung function can be caused by airway wall remodeling, bronchoconstriction, occlusion of the airway lumen by mucus and destruction of alveolar attachments of the airways within the lung (emphysema) [3]. Aberrant extracellular matrix (ECM) turnover contributes to both airway remodeling and pulmonary emphysema.

Fibroblasts play an important role in ECM turnover in the parenchyma and small airways by producing ECM constituents [4]–[6]. Transforming growth factor-β (TGF-β) is locally upregulated in COPD and is the key mediator stimulating ECM production by recruiting and activating fibroblasts and initiating their differentiation process into myofibroblasts [5], [7]–[9]. Airway fibroblasts may thus contribute to small airways remodeling in COPD. By contrast, in the peripheral lung with pulmonary emphysema, there is inadequate tissue repair and associated damage, which is perhaps due to fibroblast dysfunction [10], [11]. This discrepancy may be explained by insufficient activation of fibroblast in regions affected by emphysema to compensate for the tissue destruction by proteases. Furthermore, lung fibroblasts from patients with pulmonary emphysema show an aberrant proliferation capacity and differences in ECM synthesis [12]–[14]. Cigarette smoke can also affect a number of fibroblast functions implicated in alveolar regeneration and repair [11], [15]. Consequently, extrinsic and intrinsic dysregulation of fibroblast function in COPD along with phenotypically distinct fibroblast populations in the airways and parenchyma, may contribute to the development of both small airway fibrosis and emphysema [16], [17].

Recently, it was demonstrated that activation of the canonical WNT/β-catenin signaling pathway is associated with fibroblast activation, fibrosis and tissue repair [18], [19]. β-Catenin is an essential component of canonical WNT signaling, in which it serves a role in activating gene transcription [20]. In the presence of WNT-ligands, cytosolic β-catenin is stabilized, permitting it to serve as a transcriptional co-activator. In addition, various growth factors, including TGF-β, can activate β-catenin signaling either directly or via autocrine WNT ligand production [19], [21], [22]. Stabilized (non-phosphorylated) β-catenin activates several target genes including matrix metalloproteinases (MMP's), growth factors, ECM proteins and pro-inflammatory mediators and enzymes [23]–[31]. The role of the WNT/β-catenin pathway in COPD is largely unknown. However, in support of a role in tissue repair, a recent study indicates that activation of WNT/β-catenin signaling protects against experimental emphysema in mice [32].

In the present study, we investigated the expression of WNT-pathway genes in human lung fibroblasts and determined the functional role of the transcriptional co-activator β-catenin in regulating TGF-β_1_-induced human lung fibroblast phenotype and function. Furthermore, we compared the expression of WNT pathway genes and activation of β-catenin in primary pulmonary fibroblasts of individuals with and without COPD.

## Results

### Expression of genes required for functional WNT signaling by fibroblasts

**Figure 1:**
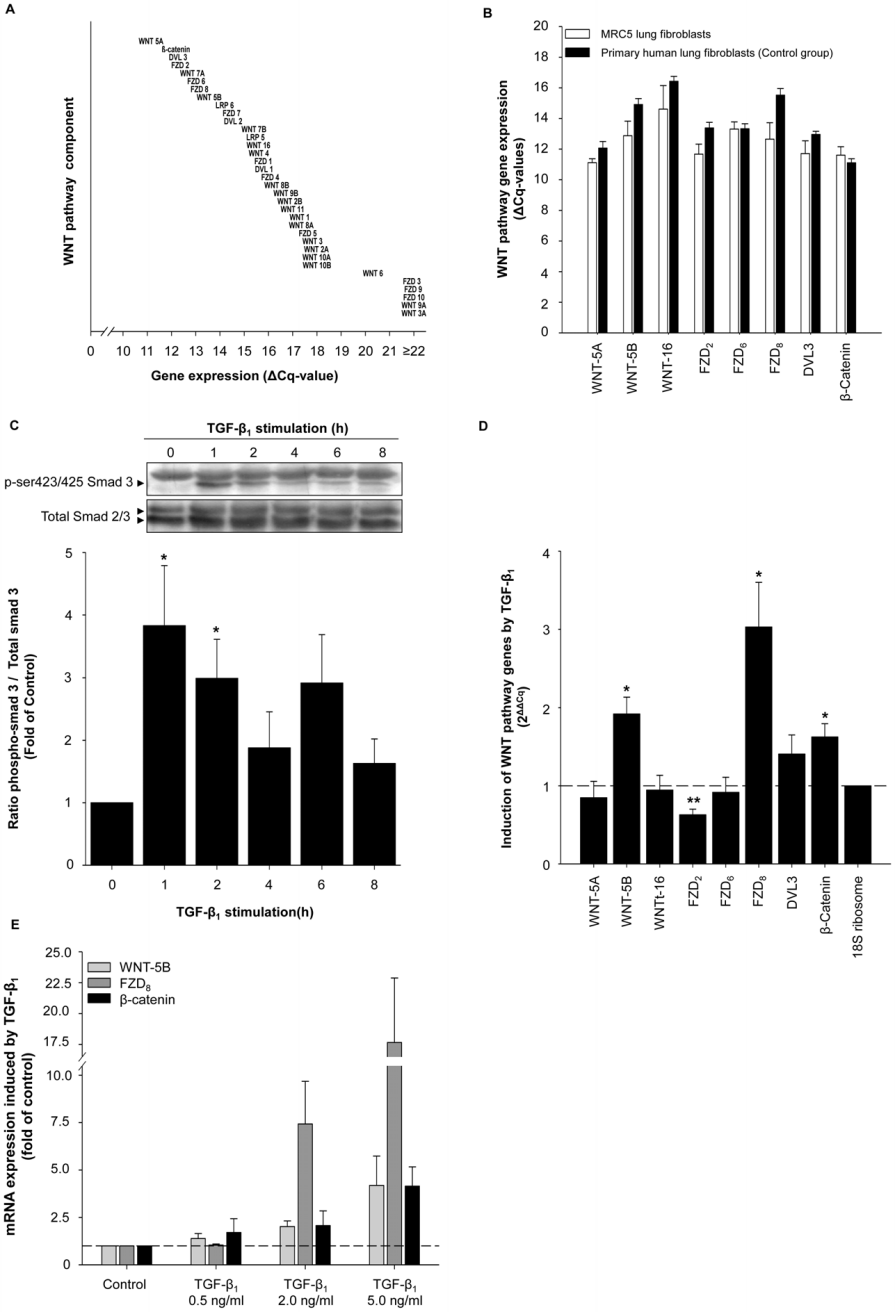
Quantitative expression of specific WNT pathway genes in human lung fibroblasts. (A) WNT pathway gene expression in MRC5 human lung fibroblasts. Data shown are average Cq-values corrected for 18s ribosomal RNA expression determined in triplicate by quantitative real-time PCR. Of note; a lower Cq-value corresponds with higher gene expression. (B) The WNT pathway genes WNT-5A, WNT-5B, WNT-16, FZD_2_, FZD_6_, FZD_8_, DVL3 and β-catenin were analyzed by quantitative real-time PCR in MRC-5 fibroblasts and primary human lung fibroblasts. (C) Time-dependent activation of smad3 in response to TGF-β_1_ (2 ng/ml). Phosphorylation of ser423/425-smad3 was evaluated in whole cell lysates by immunoblotting using specific antibodies. Equal protein loading was verified by the analysis of total smad2/3. Data represents mean ± s.e.m. of 6 independent experiments. *p<0.05 compared to untreated MRC-5 fibroblasts determined by a two-tailed student's *t*-test for paired observations. (D) qRT-PCR analysis of WNT-5A, WNT-5B, WNT-16, FZD_2_, FZD_6_, FZD_8_, DVL3 and β-catenin in MRC-5 fibroblasts after 4 h of of TGF-β_1_ (2 ng/ml) stimulation. Expression of WNT pathway genes by TGF-β_1_ is corrected for 18S rRNA and expressed relative to untreated MRC-5 fibroblasts. Data represents mean ± s.e.m. of 5 independent experiments. *p<0.05, **p<0.01 compared to untreated MRC-5 fibroblasts determined by a two-tailed student's *t*-test for paired observations. (E) Effect of increasing concentrations TGF-β_1_ on WNT-5B, FZD_8_ and β-catenin gene expression. MRC-5 fibroblasts were stimulated with 0.5, 2.0 and 5.0 ng/ml TGF-β_1_ for 24 h. WNT-5B, FZD_8_ and β-catenin expression was determined by qRT-PCR analysis, corrected for 18S rRNA and expressed relative to untreated MRC-5 fibroblasts (control). Data represents mean ± s.e.m. of 4–7 independent experiments. p<0.05 for dose-dependency of WNT-5B, FZD_8_ and β-catenin gene expression in response to TGF-β_1_ determined by a One-way ANOVA.

We first investigated WNT pathway gene expression in MRC-5 human lung fibroblasts. A clear mRNA signal was observed for the majority of WNT pathway genes, but with considerable differences in the degree of expression (Figure 1A). The WNT-ligands WNT-5A, WNT-5B and WNT-16, the Frizzled (FZD) receptors FZD_2_, FZD_6_ and FZD_8_ as well as the intracellular signaling protein dishevelled (DVL3) and the key-effector of canonical WNT signaling, β-catenin, were abundantly expressed (figure 1A and 1B). This subset of specific WNT pathway genes was selected for further studies based on their abundant expression at baseline, on previous findings indicating the regulation of these genes by TGF-β_1_ in airway smooth muscle (unpublished data), and based on recent literature indicating the involvement of the selected WNT ligands, FZD receptors and intracellular signaling molecules in cellular processes relevant for fibroblasts function [35]–[37]. A role for additional WNT pathway genes in fibroblast function can, however, not be ruled out. To investigate if these genes were also highly expressed in primary human lung fibroblasts, we performed qRT-PCR analysis of these WNT pathway genes in fibroblasts of individuals without COPD (control) and compared them to the expression in MRC-5 fibroblasts, which produced similar results (figure 1B).

Recently, studies have suggested that activation of WNT signaling plays an important role in remodeling and repair in several organs and that it may show a cooperative interaction with the TGF-β_1_/smad pathway [18], [38]–[42]. Smad signaling is key in TGF-β_1_ induced cellular responses and therefore we investigated first the phosphorylation of smad3. MRC-5 fibroblasts were stimulated with TGF-β_1_ (2 ng/ml) for various time-points resulting in a time-dependent increase in ser423/425-smad3 phosphorylation, which was most profound at the early time-points of 1–2 hours (figure 1C).

Next, we wondered if TGF-β_1_ would affect the expression of the selected WNT pathway genes in human lung fibroblasts. Stimulation of MRC-5 human lung fibroblasts with TGF-β_1_ (2 ng/ml; 4 hours) altered the expression profile of specific WNT-pathway genes (figure 1D). The expression of WNT-5B, FZD_8_ and β-catenin was significantly increased in TGF-β_1_ treated fibroblasts (fold-induction 1.92±0.22, 3.03±0.57 and 1.66±0.16, respectively), whereas FZD_2_ mRNA expression was significantly down-regulated to 0.63±0.07-fold compared with untreated fibroblasts. The expression of WNT-5A, WNT-16, FZD_6_ and DVL3 was unaltered after TGF-β_1_ stimulation (figure 1D). Concentration-response curves with 0.5, 2 and 5 ng/ml of TGF-β_1_ show that the expression of WNT-5B, FZD_8_ and β-catenin in MRC-5 fibroblasts is concentration dependent (figure 1E).

### Differential WNT pathway gene expression in primary lung fibroblasts from individuals with and without COPD

To investigate if WNT pathway gene expression was altered in COPD, we quantified the most abundant WNT signaling pathway genes by qRT-PCR in primary human lung fibroblasts from individuals with and without COPD at different stages of disease (COPD GOLD stage II or GOLD stage IV). The clinical characteristics of the subject groups are represented in table 1. The individuals with COPD stage II were significantly older and individuals with COPD stage IV had a lower body mass index (BMI). The smoking history (i.e. smoking status and pack-years) and gender distribution was similar in all groups.

**Table 1:** Clinical characteristics of the subjects involved in the studies.

	Subject groups		
	Control	COPD stage II	COPD stage IV
**Number of subjects**	7	5	6
**Age (years)**	58(46–74)	73(70–77)*	55(52–59)
Body mass index(Kg/m^2^)	26.5±2.0(n = 4)	25.5±1.5(n = 5)	21.0±0.6#(n = 6)
**Sex**			
Male	4	5	4
Female	3	0	2
**Smoking status**			
Ex-smoker	4	4	6
Current smoker	2	1	0
Non-smoker	1	0	0
**Pack-years**	36(0–70)	42.5(17.5–55)	30(20–38)
**FEV_1_ % predicted**	96.9(75.9–118.0)	52.6(38.0–66.9)	17.1***(14.0–18.5)
**FEV_1_/FVC**	76.0(71.4–81.5)	49.3(37.0–60.7)	27.7**(14.0–62.1)

All values are represented as median values with ranges in parentheses. Ex –smokers = not smoking for at least one year. FEV_1_ % predicted = Forced Expiratory Volume in 1 second as percentage of predicted value; FVC = Forced Vital Capacity. Stage means severity of COPD according to GOLD criteria. Statistical significance determined by a kruskall-wallis ANOVA followed by Dunn's multiple comparisons test or a two-way student's t-test for unpaired observations.

*p<0.05,

**p<0.01,

***p<0.001 compared to Control group and

# p<0.05 compared to individuals with COPD stage II.

#### WNT ligands

**Figure 2:**
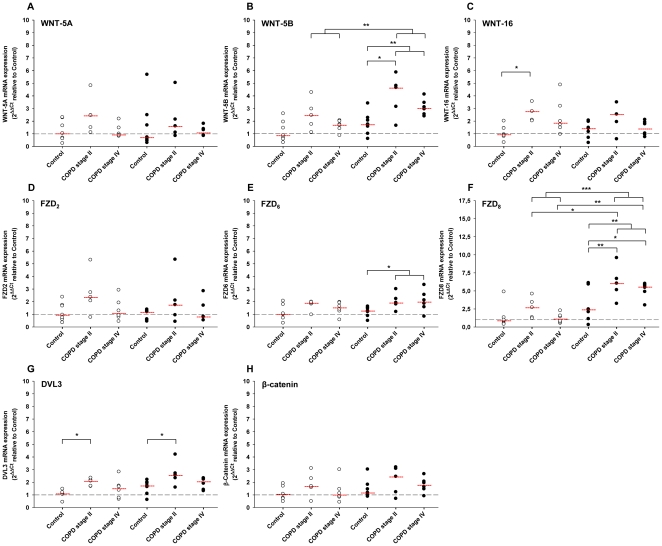
Differential WNT pathway gene expression in primary lung fibroblasts from individuals with and without COPD. Primary lung fibroblasts were isolated from individuals without (control) and with COPD (GOLD stage II and IV) as described in the materials en methods. qRT-PCR analysis of WNT-5A (A), WNT-5B (B), WNT-16 (C), FZD_2_ (D), FZD_6_ (E), FZD_8_ (F), DVL3 (G) and β-catenin (H) mRNA of primary lung fibroblasts treated with or without TGF-β_1_ (2 ng/ml) for 4 h. Expression of WNT pathway genes is plotted relative to the mean expression in untreated fibroblasts from controls. Data are derived from 7 controls and 11 COPD patients (5 GOLD stage II and 6 GOLD stage IV). mRNA expression was determined both at baseline (open circles; ○) and after TGF-β stimulation (closed circles; •). Median of each group is indicated by -----. *p<0.05, **p<0.01, ***p<0.001, two-tailed student's *t*-test for unpaired observations or a One-way ANOVA followed by a Newman-Keuls multiple comparison test.

First, we determined the expression profile of the ligands WNT-5A, WNT-5B and WNT-16. No significant differences were observed in the basal expression of WNT-5A and WNT-5B in fibroblasts from individuals with COPD compared to individuals without COPD (controls), whilst WNT-16 expression was significantly higher in individuals with COPD stage II (figure 2A–C) In line with the MRC-5 fibroblasts, stimulation with TGF-β_1_ had no effect on mRNA expression of WNT-5A and WNT-16, but induced mRNA expression of WNT-5B. Interestingly, WNT-5B mRNA expression in TGF-β_1_ treated fibroblasts was higher in individuals with than without COPD (figure 2B). The mRNA expression of WNT-5A and WNT-16 in TGF-β_1_ treated fibroblasts was not different in individuals with or without COPD (figures 2A and 2C).

#### Frizzled (FZD) receptors

Basal mRNA expression of the FZD-receptors FZD_2_, FZD_6_, and FZD_8_ was similar in fibroblasts from individuals with and without COPD, independent of GOLD stage (figure 2D–F). In contrast to what we observed in MRC-5 cells, in fibroblasts from individuals without COPD, the expression of the FZD_2_ receptors was unaltered in response to TGF-β_1_ (figure 2D–F). Likewise, the expression of FZD_2_ did not differ after TGF-β_1_ stimulation in fibroblasts from individuals with COPD, independent of GOLD stage (Figure 2D). Conversely, FZD_6_ mRNA expression in fibroblasts from individuals with COPD was upregulated in the presence of TGF-β_1_. The total FZD_6_ mRNA content was significantly higher (1.67±0.18–fold) in fibroblasts from individuals with COPD than controls after TGF-β_1_ stimulation (figure 2E). In addition, TGF-β_1_ up regulated FZD_8_ mRNA expression in fibroblasts from individuals with as well as without COPD. Total FZD_8_ mRNA content in TGF-β_1_ stimulated fibroblasts was higher in individuals with either COPD stage II or stage IV than in fibroblasts from controls (figure 2F).

#### DVL3 and β-catenin

The mRNA expression at baseline of β-catenin, the key effector of WNT signaling, was comparable in fibroblasts from individuals with and without COPD, whereas the expression of the intracellular WNT signaling protein DVL3 was significantly higher in individuals with COPD stage II (figure 2G). However, no differences in DVL3 mRNA expression were observed between controls and individuals with COPD stage IV. TGF-β_1_ stimulation resulted in an upregulation of both DVL3 and β-catenin mRNA in fibroblasts from individuals with and without COPD (figure 2G–H). After TGF-β_1_ stimulation, total DVL3 mRNA levels in fibroblasts from COPD patients with GOLD stage II were significantly higher than in controls (figure 2G).

### TGF-β_1_ induces myofibroblast differentiation and activates β-catenin signaling

**Figure 3:**
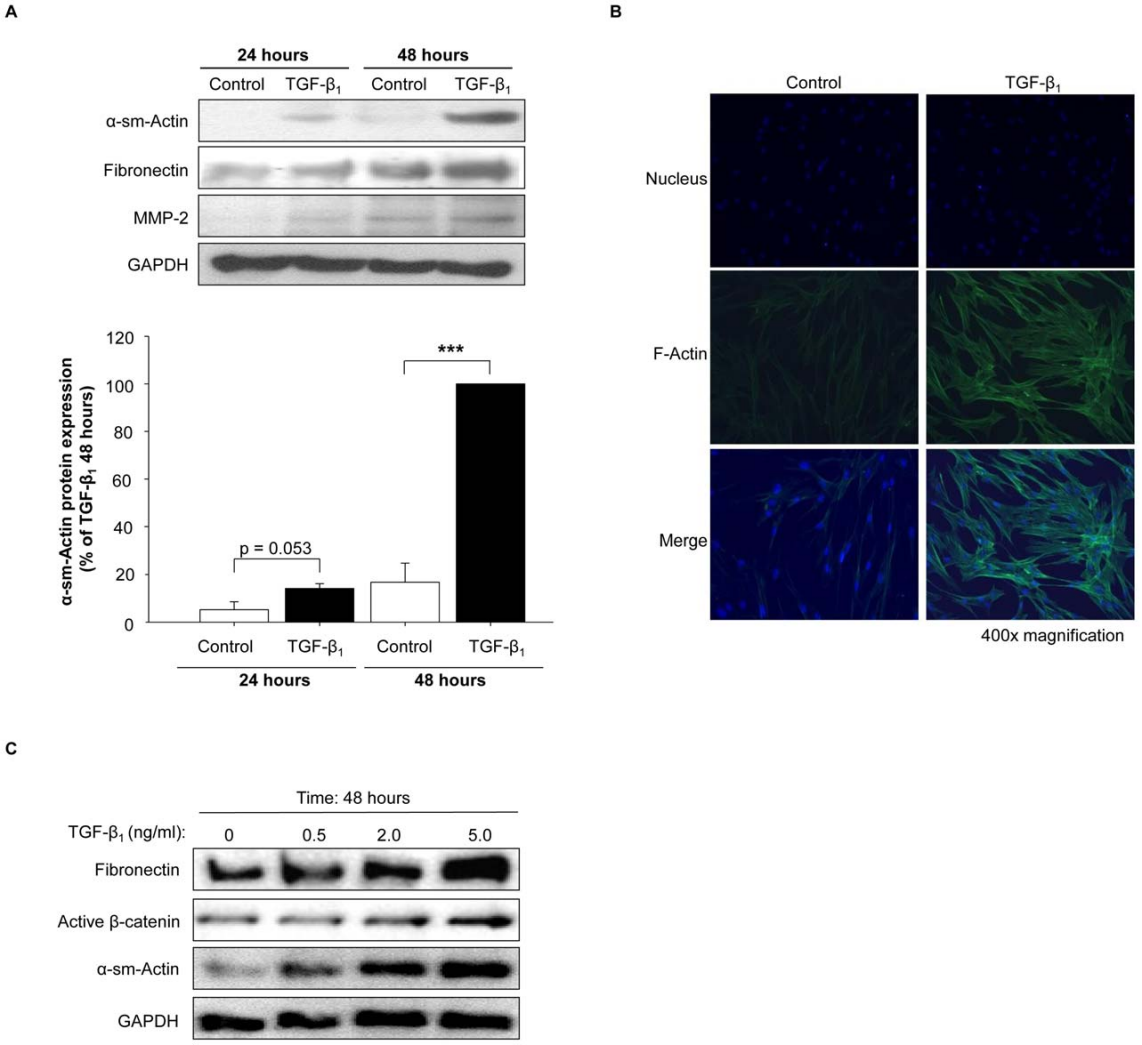
TGF-β_1_ induces myofibroblast differentiation of MRC-5 lung fibroblasts. MRC-5 fibroblasts were grown to confluence and treated for 24 h or 48 h with 2 ng/ml of TGF-β_1_. (A) Expression of the myofibroblasts markers α-sm-actin, fibronectin and MMP-2 was evaluated in whole cell lysates by immunoblotting using specific antibodies. Equal protein loading was verified by the analysis of GAPDH. Representative immunoblots of 5–8 independent experiments are shown. ***p<0.001, two-way student's *t*-test for paired observations. (B) Evaluation of stress fiber formation in MRC-5 lung fibroblasts after TGF-β_1_ stimulation. MRC-5 lung fibroblasts were treated for 48 h with TGF-β_1_ (2 ng/ml) and subsequently fixed and permeabilized. Cells were stained for filamentous actin (488 phalloidin; green) and nucleus (Hoechst 3342; blue). Pictures were taken at 400× magnification. (C) Effect of increasing concentrations TGF-β_1_ on myofibroblast differentiation. MRC-5 fibroblasts were stimulated with 0.5, 2.0 and 5.0 ng/ml TGF-β_1_ for 48 h. Expression of α-sm-actin, fibronectin and active β-catenin was evaluated in whole cell lysates by immunoblotting using specific antibodies. Equal protein loading was verified by the analysis of GAPDH. Representative immunoblots of 4 independent experiments are shown.

We next studied activation of the WNT effector β-catenin in response to TGF-β_1_ and determined its functional role in myofibroblast differentiation. Treatment of MRC-5 human lung fibroblasts for 48 hours with TGF-β_1_ (2 ng/ml) resulted in a significant increase in protein expression of the differentiation markers α-sm-actin, fibronectin and MMP-2 (figure 3A). Cytochemical staining for filamentous actin (F-actin) indicated that TGF-β_1_ (48 hours) distinctively induced the formation of stress fibers (F-actin) in these cells, another indication of myofibroblast differentiation (figure 3B). Stimulation of MRC-5 fibroblasts with 0.5, 2 and 5 ng/ml of TGF- β_1_ for 48 hours shows that the increase of fibronectin and α-sm-actin protein expression is concentration-dependent (figure 3C). Interestingly, the expression of active (unphosphorylated) β-catenin followed similar concentration dependence (figure 3C). Therefore, the activation of β-catenin in response to TGF-β_1_ was investigated in more detail.

**Figure 4:**
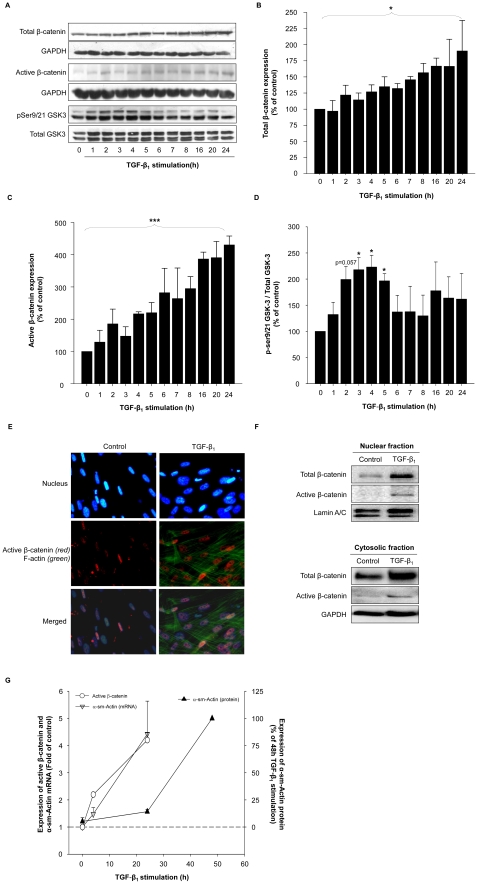
Treatment with TGF-β_1_ increases β-catenin signaling in MRC-5 lung fibroblasts. MRC-5 fibroblasts were grown to confluence and treated for up to 24 h with TGF-β_1_ (2 ng/ml). (A) Expression of total β-catenin, active (non-phosphorylated) β-catenin and ser9/21 phosphorylation of GSK-3 were evaluated by immunoblotting using specific antibodies. Equal protein loading was verified by the analysis of GAPDH or total GSK-3, respectively. Responses of TGF-β_1_ on total and active β-catenin expression (B and C) and ser9/21-GSK-3 phosphorylation (D) were quantified by densitometry, representing mean ± s.e.m. of 3 independent experiments. *p<0.05, ***p<0.001, two-tailed student's *t*-test for paired observations or repeated measures ANOVA followed by a Newman-Keuls multiple comparison test. (E) Evaluation of cellular localization of active (non-phosphorylated) β-catenin in MRC-5 lung fibroblasts stimulated with TGF-β_1_ (2 ng/ml) for 48 h. Fixed and permeabilized MRC-5 fibroblasts were (immuno)cytochemically stained for active (non-phosphorylated) β-catenin (Cy3; red) and stained for filamentous actin (488 phalloidin; green) and nucleus (Hoechst 33342; blue). Pictures were taken at 400× magnification. (F) Increased cytosolic and nuclear expression of β-catenin in response to TGF-β_1_ stimulation. MRC-5 fibroblasts were stimulated with TGF-β_1_ (2 ng/ml) for 24 h. Subsequently cytosolic and nuclear extracts were prepared. Expression of total and active (non-phosphorylated) β-catenin was evaluated by immunoblotting. Equal protein loading was verified by the analysis of GAPDH and Lamin A/C, respectively. Representative immunoblots of 4 independent experiments are shown. (G) β-Catenin activation precedes myofibroblast differentiation. Expression of active β-catenin protein (open circles; ○), α-sm-actin mRNA (grey triangles; ▾) and α-sm-actin protein (black triangles; ▴) in response to TGF-β_1_ (2 ng/ml) was determined by immunoblotting and quantitative real time PCR. Data represents mean ± s.e.m. of 3–8 independent experiments.

Treatment of fibroblasts with TGF-β_1_ (2 ng/ml) resulted in a significant, time-dependent increase in total β-catenin protein expression after 24 hours of stimulation compared to untreated fibroblasts (figure 4A–B). Interestingly, TGF-β_1_ induced an even more pronounced increase in the expression of the transcriptionally active (non-phosphorylated) β-catenin, with kinetics similar to the induction of total β-catenin (figure 4C). Glycogen synthase kinase-3 (GSK-3) is a major protein kinase involved in regulating β-catenin cellular expression and is negatively regulated by ser9 and ser21 (ser9 of GSK-3β and ser21 of GSK-3α) phosphorylation [43]. Therefore, the effect of TGF-β_1_ on GSK-3 phosphorylation was also investigated. TGF-β_1_ induced a strong inhibitory ser9/21 phosphorylation of GSK-3 (figure 4A and 4D). As would be expected, expression of the transcriptionally active (non-phosphorylated) β-catenin distinctively increased in the cytosolic and nuclear compartment after TGF-β_1_ stimulation (figure 4E and 4F). Interestingly, activation of β-catenin signaling preceded myofibroblast differentiation (figure 4G).

### Functional role for β-catenin in TGF-β_1_-induced myofibroblast differentiation

**Figure 5:**
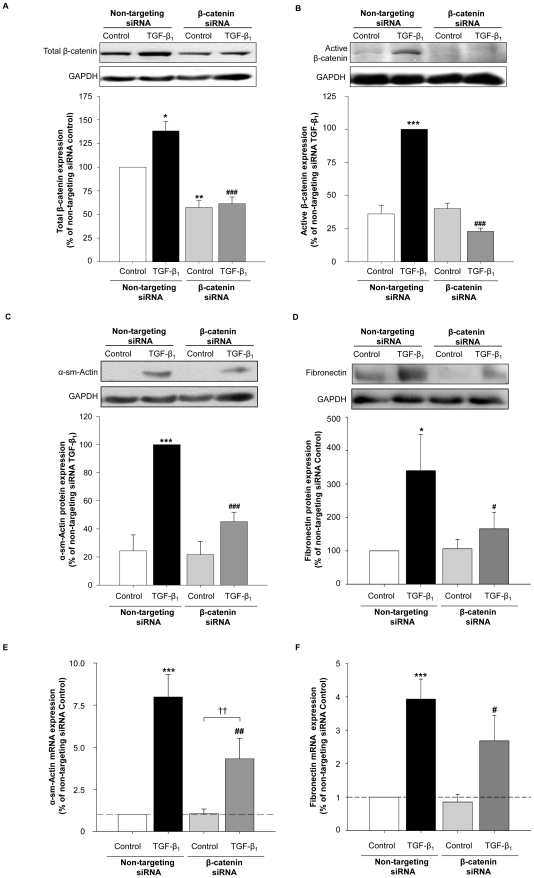
Silencing β-catenin expression by specific siRNA attenuates TGF-β_1_-induced α-sm-actin and fibronectin expression. Subconfluent MRC-5 lung fibroblast cultures were transfected with a siRNA against the β-catenin transcript. Control cultures were transfected with a non-targeting control siRNA. Transfected cells were treated with TGF-β_1_ (2 ng/ml) for 48 h. (A–B) The efficiency of β-catenin silencing was evaluated by immunoblotting the expression of (A) total β-catenin and (B) active β-catenin and GAPDH to correct for differences in protein loading. Data represent mean ± s.e.m. of 4–6 experiments. *p<0.05, **p<0.01 and ***p<0.001 compared to non-targeting siRNA control, ^###^p<0.001 compared to non-targeting siRNA treated with TGF-β_1_ determined by a one-way ANOVA followed by a Newman-Keuls multiple comparison test. (C–F) β-catenin siRNA attenuated TGF-β_1_-induced α-sm-actin (C and E) and fibronectin (D and F) gene and protein expression. Expression or mRNA was determined by real-time PCR and normalized to 18S ribosomal mRNA expression. Protein expression was determined by immunoblotting and equal protein loading was verified by the analysis of GAPDH. Responses were quantified and normalized to the expression of 18S rRNA (gene) or GAPDH (protein). Data represent mean ± s.e.m. of 5–6 independent experiments. *p<0.05, ***p<0.001 compared to non-targeting siRNA control, ^#^p<0.05, ^##^p<0.01 ^###^p<0.001 compared to non-targeting siRNA treated with TGF-β_1_, ^††^p<0.01 compared to β-catenin siRNA control, one-way ANOVA followed by a Newman-Keuls multiple comparison test.

To determine the functional role of β-catenin in myofibroblast differentiation, we used specific small interfering RNA (siRNA) to silence β-catenin protein expression. After siRNA treatment, total β-catenin expression was reduced to 57±7% in fibroblasts at baseline (figure 5A). TGF-β_1_ stimulation after siRNA transfection, resulted in a significant increase in β-catenin protein expression in the non-targeting siRNA treated fibroblasts (control) (figure 5A–B), whereas this induction of total and - more importantly - transcriptionally active β-catenin was completely abrogated in fibroblasts treated with specific siRNA against β-catenin (figure 5B).

Next, we investigated the functional effects of β-catenin silencing on TGF-β_1_-induced gene and protein expression. Non-targeting siRNA treated fibroblasts were stimulated with TGF-β_1_ for 24 and 48 hours (for mRNA and protein determination, respectively), resulting in increased expression of α-sm-actin (figure 5C and 5E) and fibronectin (figure 5D and 5F). The induction of both α-sm-actin and fibronectin was largely attenuated in fibroblasts treated with specific siRNA against β-catenin (figure 5C–E). Silencing of β-catenin expression also reduced the TGF-β_1_-induced collagen Iα1 mRNA expression, whereas the expression of plasminogen activator inhibitor-1 (PAI-1) was not affected (data not shown).

**Figure 6:**
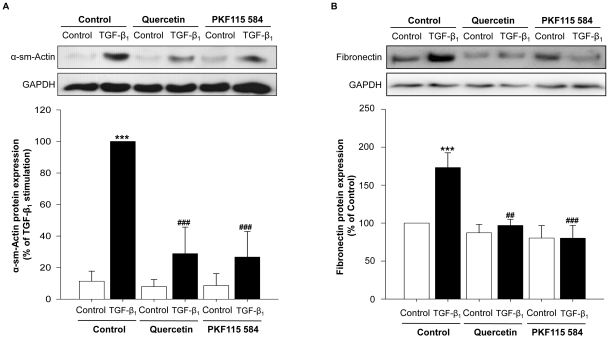
Pharmacological inhibition of β-catenin attenuates TGF-β_1_-induced α-sm-actin and fibronectin expression. Pharmacological inhibition of β-catenin/TCF_4_ signaling by quercetin or PKF115–584. Confluent MRC-5 lung fibroblasts were treated with TGF-β_1_ (2 ng/ml) for 48 h in the absence or presence of either quercetin (40 µM) or PKF115–584 (100 nM). Expression of α-sm-actin (A) and fibronectin (B) was evaluated by immunoblotting using a specific antibody. Responses were quantified by densitometry and normalized to the expression of GAPDH. Data represent mean ± s.e.m. of 3 independent experiments. ***p<0.001 compared untreated MRC-5 lung fibroblasts (control), ^##^p<0.01; ^###^p<0.001 to TGF-β_1_ treated MRC-5 lung fibroblasts determined by a one-way ANOVA followed by a Newman-Keuls multiple comparison test.

To further verify the functional role of β-catenin in lung fibroblasts we pharmacologically inhibited β-catenin signaling by either quercetin or PKF115–584, compounds that disrupt the interaction of the transcriptionally active β-catenin/T-cell factor-4 (TCF-4) complex [44]–[46]. Both pharmacological inhibitors greatly attenuated α-sm-actin induction and fully prevented the increased fibronectin deposition induced by TGF-β_1_, without affecting basal expression of either α-sm-actin or fibronectin (figure 6A–B).

### Fibroblasts of COPD patients show increased β-catenin activation and subsequent fibronectin deposition in response to TGF-β_1_

**Figure 7:**
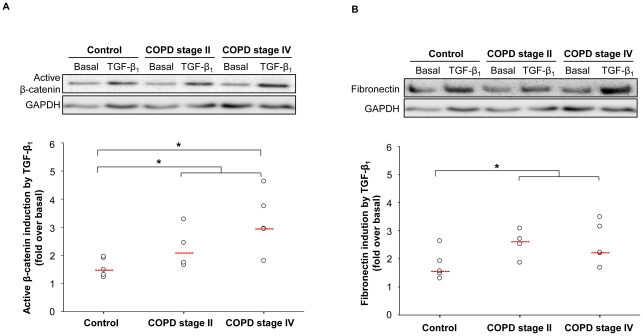
Increased β-catenin activation and fibronectin deposition in fibroblast of individuals with COPD in response to TGF-β_1_. Primary lung fibroblasts were isolated from individuals without (control) and with COPD (GOLD stage II and IV) as described in the materials en methods. The fibroblasts were grown to confluence and treated for 48 h with TGF-β_1_ (2 ng/ml). Expression of active β-catenin (A) and fibronectin (B) was evaluated by immunoblotting. Equal protein loading was verified by the analysis of GAPDH. Responses were quantified by densitometry and normalized to the expression of GAPDH. Data are derived from 5 controls and 9 COPD patients (4 GOLD stage II and 5 GOLD stage IV). Median of each group is indicated by -----. *P<0.05. Statistical differences between control and COPD were determined a two-tailed Mann-whitney test.

Fibroblasts from individuals with and without COPD had similar expression of active β-catenin at baseline. Stimulation with TGF-β_1_ also resulted in a significant induction of active β-catenin in fibroblasts from individuals with and without COPD. Interestingly, the induction of active β-catenin was significantly higher in fibroblasts from individuals with COPD than those without COPD (figure 7A). In accordance with the increase in active β-catenin, fibronectin deposition was increased after TGF-β_1_ stimulation and more so in fibroblasts from individuals with COPD (figure 7B). The expression of the myofibroblast marker α-sm-actin was also studied; however the fold induction of α-sm-actin by TGF-β_1_ treatment could not be computed as basal expression of α-sm-actin in fibroblasts was occasionally not observed. However, no significant differences were observed for the TGF-β_1_-induced α-sm-actin bands (GAPDH ratio) between individuals with and without COPD (data not shown). Thus, β-catenin activation and subsequent fibronectin deposition in response to TGF-β_1_ is enhanced in lung fibroblasts from COPD patients compared to lung fibroblasts from controls.

## Discussion

Pulmonary fibroblasts play a pivotal role in COPD by regulating ECM turnover in the lungs [4], [11]. To our knowledge, this is the first study demonstrating that WNT/β-catenin signaling in pulmonary fibroblasts may play an important role in COPD. We show that pulmonary fibroblasts express genes required for functional WNT signaling, of which WNT-5B, FZD_8_, DVL3 and β-catenin were significantly induced by TGF-β_1_ in a concentration dependent manner in both MRC-5 and primary human lung fibroblasts. Interestingly, WNT-5B, FZD_6_ and FZD_8_ expression were significantly more upregulated in response to TGF-β_1_ in primary fibroblasts from individuals with than without COPD. Furthermore, we also show that β-catenin, the key effector of canonical WNT signaling, regulates the induction of collagen1α1, α-sm-actin and fibronectin deposition by pulmonary fibroblasts in response to TGF-β_1_, whereas the expression of PAI-1 is not regulated by β-catenin. Finally, we provide evidence that the induction of transcriptionally active β-catenin and subsequent fibronectin deposition induced by TGF-β_1_ are significantly enhanced in lung fibroblasts from COPD patients.

An active and complex remodeling process is present in the peripheral lung when COPD develops, resulting in small airway fibrosis and a variable degree of emphysema. Fibroblasts are the primary cell type responsible for the production and maintenance of the extracellular matrix. Alterations in fibroblast function may therefore play an important role in COPD. In this respect, the canonical WNT/β-catenin signaling pathway is of particular interest, since this pathway has been linked to tissue repair and remodeling [20], [47]. Indeed, activation of canonical WNT/β-catenin signaling attenuates experimental emphysema in mice [32]. In that study, WNT pathway gene expression in lung tissue of COPD patients was also examined and, although the gene expression of specific WNT ligands and FZD receptors showed no notable changes in whole lung homogenate, alveolar type II cells had reduced β-catenin expression [32]. Our observations suggest that fibroblasts from the peripheral lung are more prone to TGF-β_1_ stimulation in activating WNT signaling and regulating transcription of tissue repair genes such as fibronectin, despite the fact that the peripheral lung is the primary site of tissue destruction associated with pulmonary emphysema [2]. We also demonstrate that WNT-16 and DVL3 expression is higher at baseline in fibroblasts of individuals with COPD stage II, although curiously this did not result in increased baseline expression of either total or active β-catenin. Of interest is that this intrinsic difference in WNT pathway activation was seen for fibroblasts from both GOLD stage II and IV COPD patients compared to fibroblasts from controls.

We propose that during COPD pathogenesis, irrespective of GOLD stage, fibroblasts from the peripheral lung are promoted to repair tissue damage, but that this repair response becomes insufficient in more advanced stages of disease. This can either be because of the development of intrinsic defects in the lung fibroblast (e.g. reduced proliferative capacity of fibroblasts in advanced stages of COPD [12] or altered intracellular Smad signaling [10]) or because of aberrant activation of fibroblasts by the locally expressed (pro-inflammatory) cytokines in the lung and / or the presence of cigarette smoke [5], [11], [48]. As a result, the destructive response in the lung may proceed and the tissue repair response by fibroblasts is deviant and not adequate. In line with this hypothesis the relative production of the proteoglycan versican as well as the expression of the pro-inflammatory enzyme cyclooxygenase-2 (COX-2), both direct targets of WNT/β-catenin signaling [29], [49], [50], are higher in parenchymal fibroblasts from COPD patients than controls [15], [17]. Furthermore, versican expression is increased in pulmonary alveolar parenchyma of mild to moderate emphysematous COPD patients and is negatively correlated with FEV_1_ [51]. These findings suggest that in COPD, parenchymal fibroblasts may have activated canonical WNT signaling, also in regions affected by emphysema, which regulates the subsequent synthesis of specific ECM components and enzymes.

The smoking histories (pack-years and smoking status) of the individuals with and without COPD were very similar, thus excluding that the observed differences between COPD and controls were primarily due to differences in smoking habits. No separate non-smoking control group (never smokers) was included in the study design. Therefore, this study does not provide insight into the effect of smoking on WNT pathway gene expression by fibroblasts. There is a statistically significant difference in age between individuals with COPD stage II (older) and individuals with either COPD stage IV or no COPD. Since the baseline expression of WNT pathway genes is higher in fibroblasts from COPD stage II compared to the other two groups (significant for WNT-16 and DVL3), we investigated the correlation between WNT pathway gene expression and age in the individuals without COPD (figure S2). We did not observe any significant correlation between age and WNT pathway gene expression, implying that these differences in gene expression at baseline are not primarily due to age.

Tissue repair by fibroblasts is a complex process involving the interplay of various growth factors and intracellular signaling pathways. Recently, crosstalk between WNT signaling pathway and growth factors in fibroblasts has been demonstrated [19], [52]. For instance, the WNT ligands WNT-3A and WNT-10B activate β-catenin signaling in NIH 3T3 fibroblasts resulting in an increased mRNA expression of connective tissue growth factor (CTGF, CCN2), endothelin-1 and TGF-β [53]. Moreover, the expression of WNT1-inducible signaling protein-1 (WISP-1), a member of the CCN family of secreted cysteine-rich matricellular proteins and a direct target gene of WNT signaling, is increased in patients with idiopathic pulmonary fibrosis (IPF) and contributes to disease pathogenesis [41]. In dermal fibroblasts, both EGF and TGF-β increased β-catenin protein stability and induced β-catenin-mediated TCF-dependent transcriptional activity [19]. Current literature is limited concerning the signaling pathways involved in the regulation of WNT pathway gene expression by TGF-β. However, recently it was suggested that WNT gene expression may be regulated by smad proteins, as dickkopf (DKK) and casein kinase 1 (CSNK1A1) were predicted smad targets genes [54]. In the present study, we show that specific WNT genes are upregulated after 4 hours of TGF-β_1_ treatment, which corresponds with the kinetics of activation of smad3. In addition, other signaling pathways such as ERK1/2 may be activated by TGF-β_1_ that regulate the WNT pathway gene expression as demonstrated for β-catenin in our recent report [55]. Clearly, future studies are required to characterize the regulation of WNT pathway gene expression by TGF-β_1_ in more detail, as we report that TGF-β_1_ induces WNT and FZD mRNA expression in human lung fibroblasts and activates β-catenin signaling, which contributes to the fibroblast phenotype and function.

Our data suggest that TGF-β_1_ induces β-catenin expression via several intracellular mechanisms. β-Catenin levels are tightly regulated by the constitutively active enzyme GSK-3. A fraction of cellular GSK-3 forms a complex with AXIN, casein kinase I (CK-I) and adenomatous polyposis coli (APC); this complex phosphorylates and subsequently targets β-catenin for proteosomal degradation [20]. The activity of both GSK-3 isoforms (e.g. GSK-3α and GSK-3β) is negatively regulated by serine (ser9 and ser21 of GSK-3β and GSK-3α, respectively) phosphorylation, which can be induced by numerous stimuli, including growth factors [21], [43], [55], [56]. We demonstrate that TGF-β_1_ induces a transient time-dependent phosphorylation of both GSK-3 isoforms in MRC-5 fibroblasts, which might account for the initial increase of β-catenin stability. In addition, enhanced secretion of canonical WNT ligands by fibroblasts in response to TGF-β_1_ may signal in an autocrine fashion, which then stabilizes β-catenin by disrupting the GSK-3/AXIN/CK-I/APC complex [20]. The increase in β-catenin protein expression after TGF-β_1_ stimulation progresses even when GSK-3 phosphorylation has returned to basal levels, supporting such an autocrine signaling loop. In addition, β-catenin protein expression can be induced by growth factors by *de novo* synthesis of the protein [55]. The underlying mechanisms by which TGF-β_1_ induces β-catenin expression in pulmonary fibroblasts are therefore not fully understood and require further exploration.

Surprisingly, TGF-β_1_ did not or only modestly affect the mRNA expression of the canonical WNT target genes dickkopf-1 (DKK-1), vascular endothelial growth factor (VEGF), interleukin-8 (IL-8) or MMP-2, and attenuated AXIN-2 mRNA expression in MRC-5 fibroblasts (figure S3), even though nuclear β-catenin was clearly induced. Thus, β-catenin contributes to the transcriptional activity induced by TGF-β_1_, but this transcriptional activity may be different from that induced by canonical WNT ligands. Indeed, WNT-3A activates β-catenin signaling in fibroblasts but does not or very modestly activate the transcription of collagen-1 and α-sm-actin, whereas it potentiates the effect of TGF-β on these myofibroblasts markers [57], [58]. This implies that the interaction between TGF-β_1_/smad and WNT/β-catenin signaling directs transcription to specific genes, which may be different from those activated by canonical WNT stimulation alone. In support, it was recently demonstrated smad proteins and β-catenin can directly interact, thereby (synergistically) activating the transcription of specific genes [27], [57]. Thus, further exploration of crosstalk between growth factors, in particular TGF-β_1_, and the WNT-signaling pathway in lung fibroblasts is of major interest to understand tissue repair mechanisms.

Our results further show that TGF-β_1_ induced collagen Iα1 and PAI-1 mRNA as well as α-sm-actin and fibronectin protein expression, indicative of fibroblast activation [59]. This activation of fibroblasts was accompanied by an increased expression of transcriptionally active β-catenin, which was primarily present in the nuclei of the fibroblasts. Silencing of β-catenin as well as pharmacological inhibition of β-catenin by either quercetin or PKF115-584, compounds that interrupt the β-catenin/TCF4 interaction [44]–[46], greatly attenuated the TGF-β_1_-induced collagen Iα1, α-sm-actin and fibronectin expression. However, the induction of PAI-1 did not change, indicating that β-catenin directs TGF-β_1_ signaling to specific intracellular pathways. Further, these data indicate that the responsiveness of the fibroblasts to TGF-β_1_ was not affected by down regulation of β-catenin. Collectively, these data demonstrate that β-catenin signaling plays an important role in the activation process of pulmonary fibroblasts. This may contribute to COPD pathogenesis, because the activation of β-catenin signaling and subsequent fibronectin deposition in response to TGF-β_1_ is higher in lung fibroblasts from patients with than without COPD.

In conclusion, our results indicate that the WNT/β-catenin signaling pathway is activated in pulmonary fibroblasts in response to the cytokine TGF-β_1_. In primary fibroblasts of COPD patients, this activation is greatly enhanced compared to healthy controls, as is the induction of β-catenin. This suggests that WNT/β-catenin signaling plays an important role in tissue repair in the lung, and that targeting β-catenin-dependent gene transcription holds promise as a therapeutic intervention in COPD.

## Materials and Methods

### Ethics statement

The study protocol was consistent with the Research Code of the University Medical Center Groningen (http://www.rug.nl/umcg/onderzoek/researchcode/index) and national ethical and professional guidelines (“Code of conduct; Dutch federation of biomedical scientific societies”; htttp://www.federa.org).

### Subjects

Primary lung fibroblasts were cultured from lung tissue obtained from 18 individuals with and without COPD. Classification of COPD severity was based on the Global initiative for chronic obstructive lung disease (GOLD) criteria [1]. Fibroblasts obtained from these individuals, were divided into three categories: fibroblasts from individuals with moderate (GOLD stage II, n = 5), and severe COPD (stage IV, n = 6), and from individuals with histologically normal lungs (n = 7). Emphysema was assessed by routine histological examination of lung tissue, which was performed by an experienced pulmonary pathologist (WT). Fibroblasts were isolated from peripheral lung tissue of which areas with no macroscopically visible airways and blood vessels were used. Clinical characteristics of the groups are presented in table 1.

Tissue from the control group (median forced expiratory volume in one second (FEV_1_) 96.9% predicted) was derived from noninvolved lung tissue of patients undergoing surgical resection for pulmonary carcinoma. Patients had no airway obstruction and no chronic airway symptoms, such as cough and sputum production. Material was always taken as far away as possible from the tumour, or from a noninvolved lobe. No histopathological lesions were present.

Tissue of GOLD stage II COPD patients (median FEV_1_ 52.6% of predicted) was derived from noninvolved lung tissue from patients undergoing resection surgery for pulmonary carcinoma. Histopathologically emphysematous lesions were present, however, of limited and varying severity. Moderate forms of emphysema can be histopathologically demonstrated by finding isolated or free-lying segments of viable alveolar septal tissue or isolated cross sections of pulmonary vessels.

Tissue of GOLD stage IV COPD patients (median FEV_1_ 17.1% predicted) was obtained from patients with COPD undergoing surgery for lung transplantation or lung volume reduction. All individuals had quitted smoking for at least 1 year before surgery. The resected tissue showed both macroscopically and microscopically severe emphysematous lesions, often accompanied by bullae. Subpleural fibrous areas were avoided.

Pulmonary fibroblast cultures were established from parenchymal lung tissue by means of an explant technique. Absence of mycoplasma contamination in the fibroblast cultures was confirmed with a mycoplasma detection kit (Roche Diagnostics, Almere, The Netherlands). Isolated cells were characterized as fibroblasts by morphological appearance and expression pattern of specific proteins [12]. All cells exhibited a characteristic staining pattern for vimentin, fibronectin, and the fibroblast marker prolyl-4-hydroxylase and lacked immunoreactivity for keratin. Five percent or less of the cells was positive for desmin and α-sm-actin.

### Cell culture

MRC-5 lung fibroblasts [33] (ATCC CCL 171) and primary lung fibroblasts from individuals with and without COPD, were cultured in Ham's F12 medium supplemented with 10% (v/v) foetal bovine serum (FBS), 2 mM L-glutamine, 100 µg/l streptomycin and 100 U/ml penicillin. Unless otherwise specified, for each experiment cells were grown to confluence and subsequently culture medium was substituted with Ham's F12 medium supplemented with 0.5% (v/v) FBS, 2 mM L-glutamine, 100 µg/l streptomycin and 100 U/ml penicillin for a period of 24 hours. Cells were stimulated for different time-points with TGF-β_1_ in Ham's F12 medium supplemented with 0.5% FBS, L-glutamine and antibiotics. When applied, pharmacological inhibitors (i.e. quercetin 40 µM or PKF115–584 100 nM) were added 30 minutes before the addition of TGF-β_1_.

### mRNA isolation and real-time PCR analysis

Total mRNA was extracted using the RNeasy mini kit (Qiagen, Venlo, The Netherlands). Briefly, cells were harvested in RNA*later* stabilization buffer and homogenized by passing the lysate 10 times through a 20 gauge needle. Lysates were then mixed with an equal volume of 70% ethanol, and total mRNA was purified using RNeasy mini spin columns. The eluted mRNA was quantified using spectrophotometry (Nanodrop, ThermoScientific, Wilmington, USA). Equal amounts of total mRNA (1 µg) were then reverse transcribed and stored at -20°C until further use.

cDNA was subjected to real-time PCR, which was performed with a MyiQ™ Single-Color detection system (Bio-Rad laboratories Inc. Life Science group, Hercules, CA, USA). In short, 12.5 µl iQ™ SYBR Green Supermix, containing fluorescein to account for well to well variation, 0.1 µM of gene-specific forward and reverse primer and 1 µl of 1∶5 diluted cDNA sample were used in a total volume of 25 µl and added to a 96 well plate. The sequences of the primers used for determining WNT pathway components and WNT target genes are listed in the supporting information tables (table S1, S2, S3, S4).

Real-time PCR data were analyzed using the comparative cycle threshold (Cq: amplification cycle number) method. Cycle parameters were: denaturation at 94°C for 30 seconds, annealing at 60°C for 30 seconds, and extension at 72°C for 30 seconds for 40 cycles followed by 5 minutes at 72°C. The amount of target gene was normalized to the endogenous reference gene 18S ribosomal RNA (Cq_gene of interest_ – Cq_18S rRNA_; designated as ΔCq). Several housekeeping genes, including β2-microglobulin (B2M; NM_00408) and phospholipase A2 (YWAHZ; NM_003406), were tested for the influence of the experimental procedure on the expression [34]. The expression of both ribosomal protein S18 (18S rRNA) and β2-microglobulin was stable in the tested conditions. Phospholipase A2 (YWAHZ; NM_003406) expression fluctuated after TGF-β stimulation, however. Ribosomal protein S18 was chosen as most optimal household gene because gene expression was most stable under basal as well as stimulation conditions (figure S1). Relative differences in gene expression were determined using the equation 2^−(ΔΔCq)^.

### siRNA transfection

MRC-5 fibroblasts were grown to ∼90% confluence in 6-well cluster plates and transiently transfected with a 21-bp, double-stranded siRNA targeted against the β-catenin transcript (Qiagen, Venlo, The Netherlands). Cells were transfected in serum-free Ham's F12 without any supplements using 1.5 µg/ml of siRNA in combination with lipofectamine 2000 transfection reagent. Control transfections were performed using a non-silencing control siRNA (Qiagen, Venlo, The Netherlands). After 6 hours of transfection, cells were washed once with warm (37°C) Hank's Balanced Salt Solution (HBSS; composition [mg/l]: KCl 400, KH_2_PO_4_ 60, NaCl 8000, NaHCO_3_ 350, Na_2_HPO_4_.1H_2_O 50, glucose 1000, pH: 7.4) followed by a period of 24 hours in Ham's F12 supplemented with 0.5% FBS, L-glutamine and antibiotics. Consecutively, medium was refreshed and cells were stimulated with TGF-β_1_ (2 ng/ml) for 48 hours.

### Preparation of cell lysates

To obtain whole cell lysates, cells were washed once with ice-cold (4°C) HBSS then lysed in ice-cold sodiumdodecylsulphate (SDS) buffer (composition: 62.5 mM Tris, 2% w/v SDS, 1 mM NaF, 1 mM Na_3_VO_4_, 10 µg/ml aprotinin, 10 µg/ml leupeptin, 7 µg/ml pepstatin A, pH 6.8). Lysates were then sonicated and protein concentration was determined according to Pierce protein determination according to the manufacturer's instructions. Lysates were stored at −20°C till further use.

### Nuclear extracts

Confluent MRC-5 fibroblasts were serum deprived in Ham's F12 medium supplemented with 0.5% (v/v) FBS, 2 mM L-glutamine, 100 µg/l streptomycin and 100 U/ml penicillin for a period of 24 hours. Subsequently cells were stimulated for 24 hour with TGF-β_1_ (2 ng/ml) and nuclear extracts were prepared using the nuclear extract kit (40010, Active Motif) according to the manufacturer's instructions. Protein concentration in the nuclear extracts was determined according to the Bradford protein assay. Nuclear extracts were stored at −80°C till further use.

### Western blot analysis

Equal amounts of protein (10–20 µg/lane) were subjected to electrophoresis on polyacrylamide gels, transferred to nitrocellulose membranes and analyzed for the proteins of interest using specific primary and HRP-conjugated secondary antibodies. By using enhanced chemiluminescence reagents, bands were either subsequently visualized on film or recorded in the G:BOX iChemi gel documentation system equipped with GeneSnap image acquisistion software (Syngene; Cambridge; UK). Band intensities were quantified by densitometry using Totallab™ software (Nonlinear dynamics; Newcastle, UK) or GeneTools analysis software (Syngene; Cambridge; UK), respectively.

### Immunocytochemistry

Lung fibroblasts were plated onto Lab-Tek™ borosilicate chamber slides and treated with TGF-β_1_ 2 ng/ml for 48 hours, fixed for 15 min at 4°C in cytoskeletal (CB) buffer (10 mM MES, 150 mM NaCl, 5 mM EGTA, 5 mM MgCl_2_ and 5 mM glucose at pH 6.1) containing 3% paraformaldehyde (PFA). Cells were then permeabilized by incubation for 5 min at 4°C in CB buffer containing 3% PFA and 0.3% Triton X-100. For immunofluorescence microscopy, fixed cells were first blocked for 2 hours at room temperature in Cyto-TBS buffer (20 mM Tris base, 154 mM NaCl, 2.0 mM EGTA and 2.0 mM MgCl_2_ at pH 7.2) containing 1% bovine serum albumin (BSA) and 2% normal donkey serum. Incubation with primary antibody (i.e unphosphorylated-β-catenin, diluted 1∶200) occurred overnight at 4°C in Cyto-TBS containing 0.1% Tween 20 (Cyto-TBST). Incubation with Cy3-conjugated secondary antibody was for 2 h at room temperature in Cyto-TBST. Filamentous actin was stained with Alexa Fluor 488 phalloidin (15 minutes at RT) and nuclei with Hoechst 3342. After staining, coverslips were mounted using ProLong Gold antifade reagent (Invitrogen) and analyzed by using an Olympus AX70 microscope equipped with digital image capture system (ColorView Soft System with Olympus U CMAD2 lens).

### Antibodies and reagents

Mouse anti-α-sm-actin, horseradish peroxidase (HRP)-conjugated goat anti-mouse antibody, HRP-conjugated goat anti-rabbit antibody and HRP-conjugated rabbit anti-goat antibody were purchased from Sigma (St. Louis, MO, USA). Goat anti-MMP-2 antibody was purchased from R&D systems (Minneapolis, MN, USA). Rabbit anti-GSK-3 antibody, goat anti-fibronectin (C20) antibody, Mouse anti-Lamin A/C antibody, mouse anti-glyceraldehyde-3-phosphate dehydrogenase (GAPDH) antibody and Rabbit-anti-Smad2/3 antibody were obtained from Santa Cruz Biotechnology (Santa Cruz, CA, USA). Rabbit anti-phospho-Ser9/21-GSK-3 antibody and rabbit anti-phospho-432/425-smad3 antibody were from Cell Signaling Technology (Beverly, MA, USA). Mouse anti-total β-catenin antibody was from BD Biosciences (San Jose, CA, USA). Mouse anti-non-phosphorylated-β-catenin antibody (clone 8E7) was from Millipore (Amsterdam, the Netherlands). Cy3 conjugated secondary antibodies were obtained from Jackson Immunoresearch (West Grove, PA, USA). Lipofectamine 2000 transfection reagent and alexa Fluor 488 phalloidin were from Invitrogen (Paisley, UK). Recombinant human TGF-β_1_ was from R&D systems (Abingdon, UK). All other chemicals were of analytical grade.

## Supporting Information

Figure S1 **18S ribosomal RNA abundance in primary fibroblasts individuals without and with COPD.** Primary lung fibroblasts were isolated from individuals without (control) and with COPD (GOLD stage II and IV) as described in the methods. The fibroblasts were grown to confluence and treated for 4 hours with TGF-β_1_ (2 ng/ml). Analysis of 18S ribosomal RNA is performed by qRT-PCR analysis with 0.025 µg of cDNA as input. (A) Average 18S rRNA expression in primary human lung fibroblasts and (B) raw Cq-values for all the individual subjects. 18S rRNA expression at baseline is indicated by open circles (○) and after TGF-β_1_ stimulation (2 ng/ml; 4 h) by closed circles (•). Median of each group is indicated by **-----**.
(TIF)

Figure S2 **No age-dependent effects on WNT pathway gene expression in pulmonary fibroblasts of individuals without COPD.** Expression of WNT-5B, DVL3 and FZD_8_ as a function of age of the individual primary lung fibroblasts isolated from individuals without COPD (control) as described in the methods. The fibroblasts were grown to confluence and subsequently mRNA was isolated. Analysis of WNT pathway gene expression is performed by qRT-PCR and corrected for 18S rRNA expression. The uninterrupted line indicates the linear regression.
(TIF)

Figure S3 **Effect of TGF-β stimulation on canonical WNT target genes in human lung fibroblasts.** qRT-PCR analysis of α-sm-actin (positive control), AXIN-2, vascular endothelial growth factor (VEGF), dickkopf-1 (DKK-1), interleukin-8 (IL-8) and matrix metalloproteinase-2 (MMP-2) in MRC-5 fibroblasts after 24 h of TGF-β_1_ (2 ng/ml) stimulation. Expression of canonical WNT target genes by TGF-β_1_ is corrected for 18S rRNA and expressed relative to untreated MRC-5 fibroblasts (control). Data represents mean ± s.e.m. of 5–10 independent experiments. *p<0.05, ***p<0.001 compared to untreated MRC-5 fibroblasts (two-tailed student's *t*-test for paired observations).
(TIF)

Table S1 **Primers used for determination of WNT ligands by qRT-PCR analysis.**
(DOCX)

Table S2 **Primers used for determination of the dishevelled protein family by qRT-PCR analysis.**
(DOCX)

Table S3 **Primers used for determination of FZD receptors by qRT-PCR analysis.**
(DOCX)

Table S4 **Primers used for determination of LRP-coreceptors, WNT target genes and housekeeping genes by qRT-PCR analysis.**
(DOCX)
