# Supplementary figures and images for: Activation of WNT / β-Catenin Signaling in Pulmonary Fibroblasts by TGF-β1 Is Increased in Chronic Obstructive Pulmonary Disease
Source: PLoS One. 2011 Sep 30;6(9):e25450. doi: 10.1371/journal.pone.0025450 (PMC3184127; doi:10.1371/journal.pone.0025450)

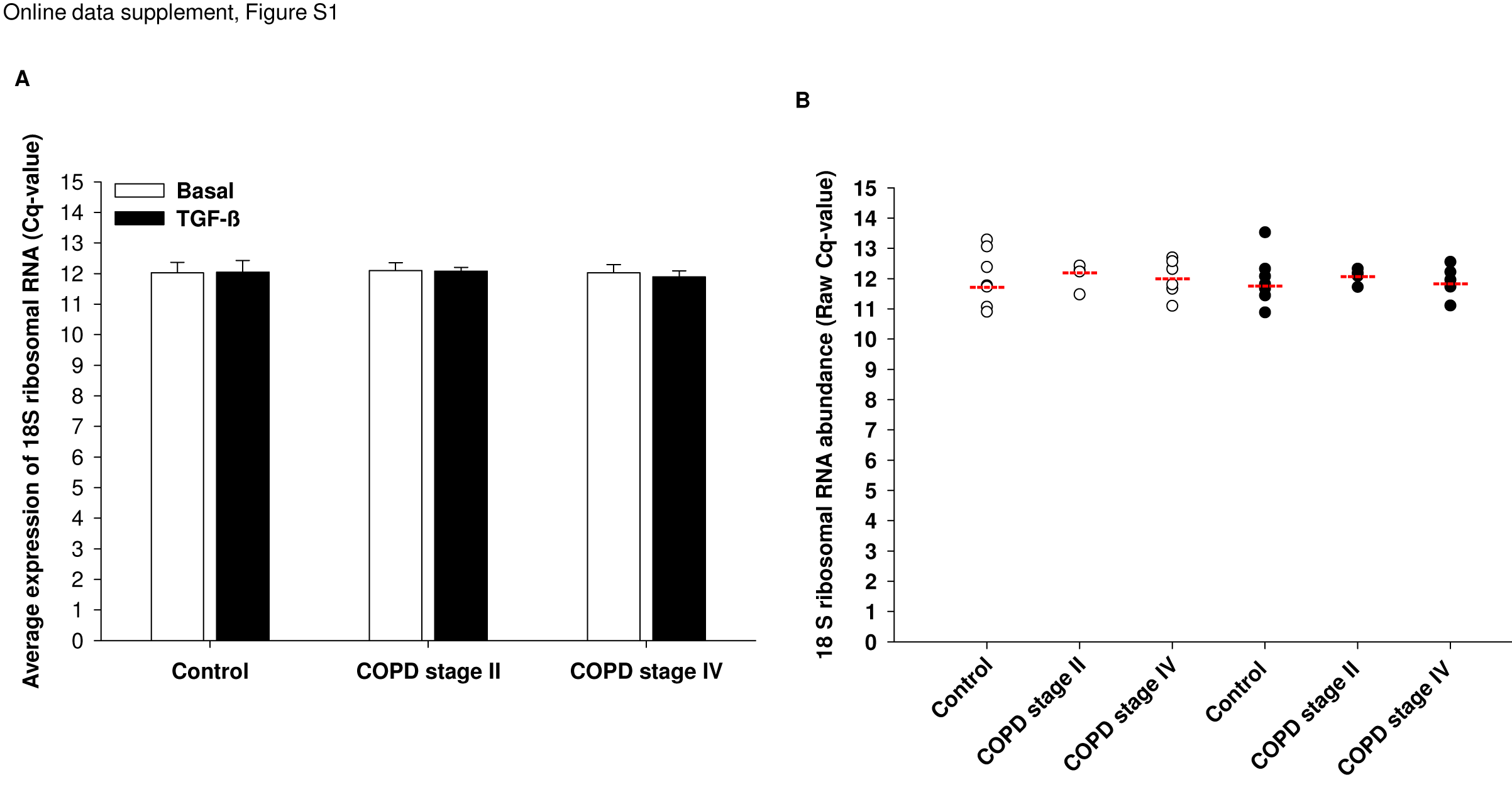

Supplement: Figure S1 — 18S ribosomal RNA abundance in primary fibroblasts individuals without and with COPD. Primary lung fibroblasts were isolated from individuals without (control) and with COPD (GOLD stage II and IV) as described in the methods. The fibroblasts were grown to confluence and treated for 4 hours with TGF-β1 (2 ng/ml). Analysis of 18S ribosomal RNA is performed by qRT-PCR analysis with 0.025 µg of cDNA as input. (A) Average 18S rRNA expression in primary human lung fibroblasts and (B) raw Cq-values for all the individual subjects. 18S rRNA expression at baseline is indicated by open circles (○) and after TGF-β1 stimulation (2 ng/ml; 4 h) by closed circles (•). Median of each group is indicated by -----. (TIF) [file pone.0025450.s001.tif]

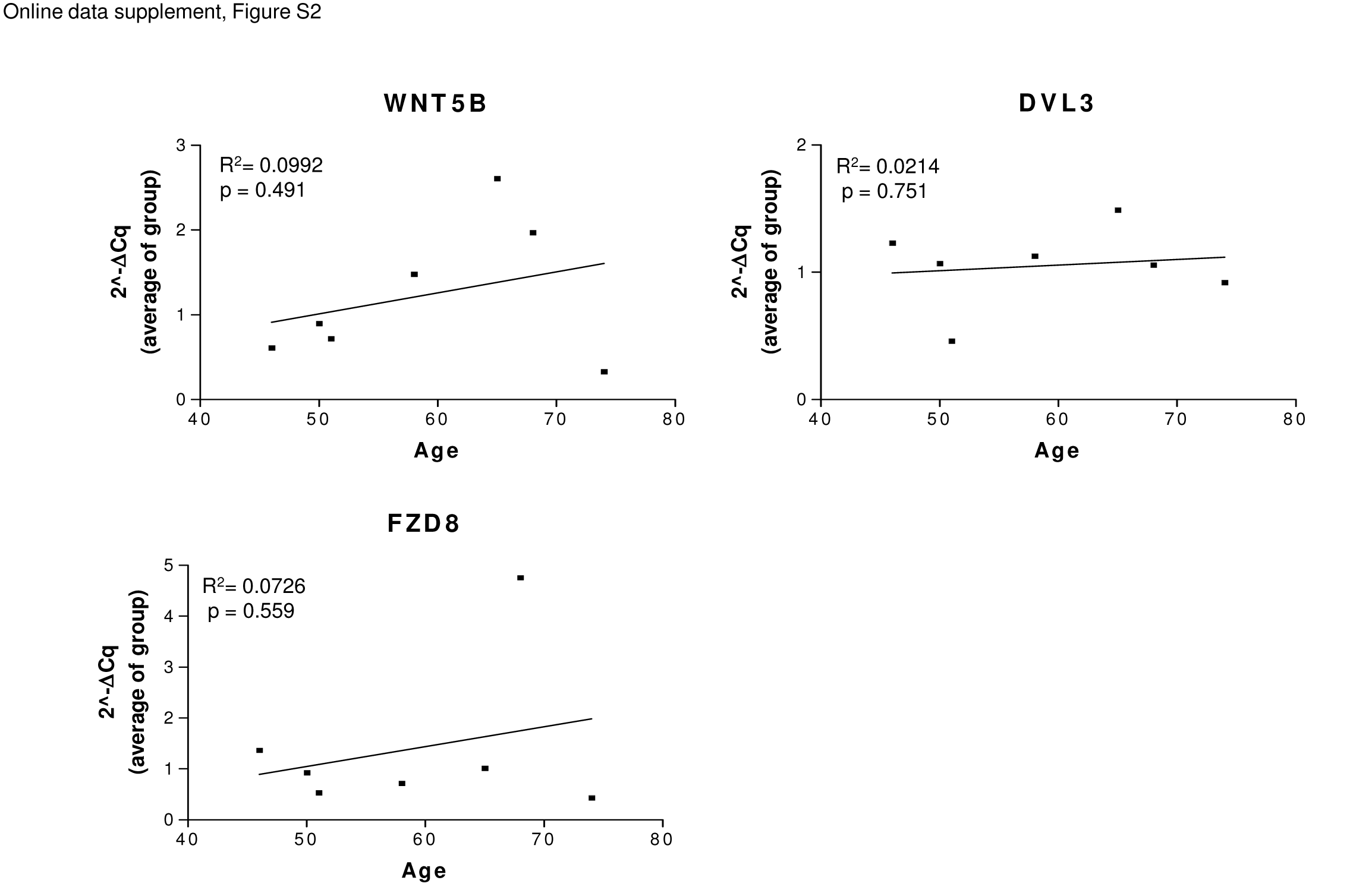

Supplement: Figure S2 — No age-dependent effects on WNT pathway gene expression in pulmonary fibroblasts of individuals without COPD. Expression of WNT-5B, DVL3 and FZD8 as a function of age of the individual primary lung fibroblasts isolated from individuals without COPD (control) as described in the methods. The fibroblasts were grown to confluence and subsequently mRNA was isolated. Analysis of WNT pathway gene expression is performed by qRT-PCR and corrected for 18S rRNA expression. The uninterrupted line indicates the linear regression. (TIF) [file pone.0025450.s002.tif]

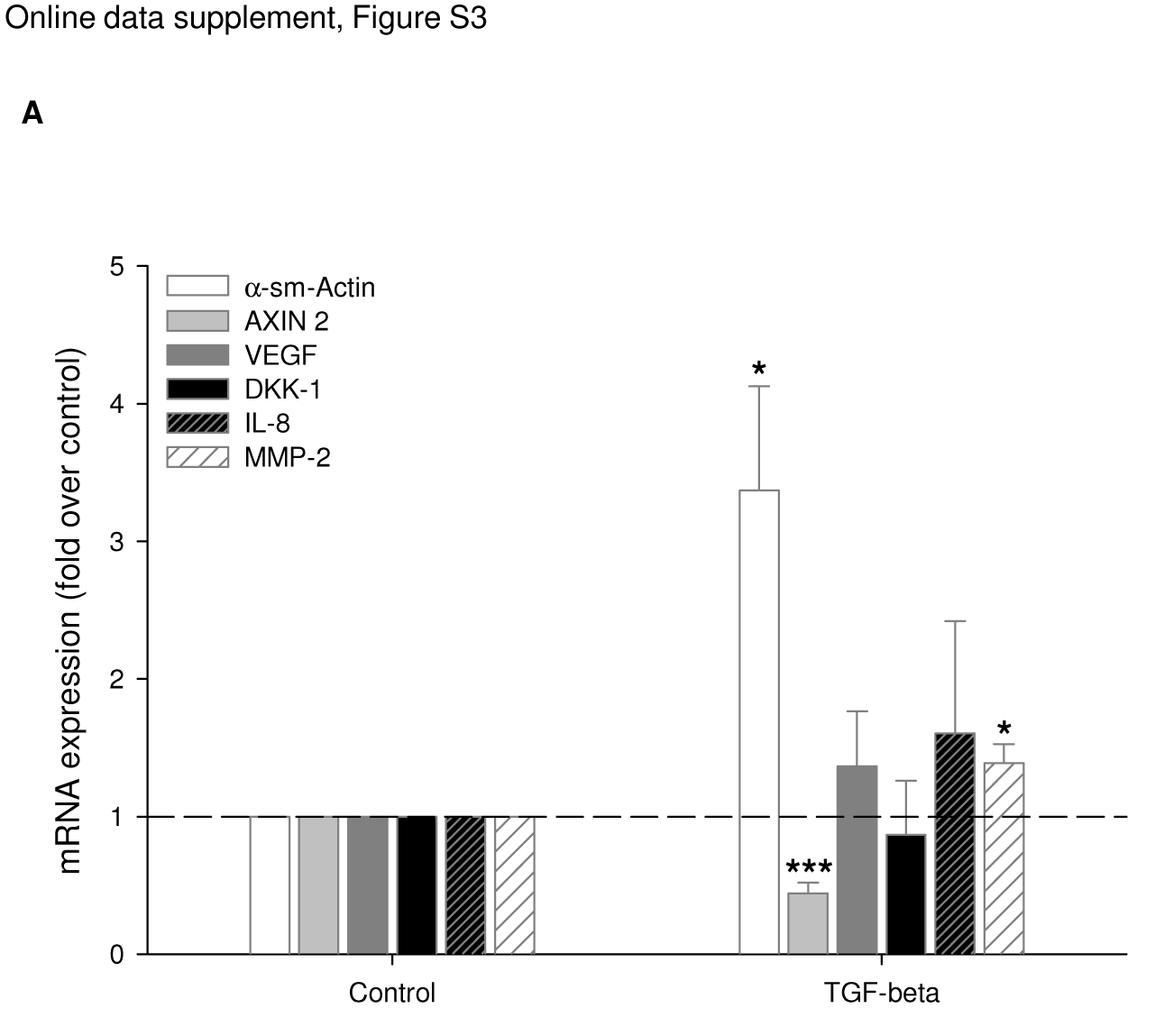

Supplement: Figure S3 — Effect of TGF-β stimulation on canonical WNT target genes in human lung fibroblasts. qRT-PCR analysis of α-sm-actin (positive control), AXIN-2, vascular endothelial growth factor (VEGF), dickkopf-1 (DKK-1), interleukin-8 (IL-8) and matrix metalloproteinase-2 (MMP-2) in MRC-5 fibroblasts after 24 h of TGF-β1 (2 ng/ml) stimulation. Expression of canonical WNT target genes by TGF-β1 is corrected for 18S rRNA and expressed relative to untreated MRC-5 fibroblasts (control). Data represents mean ± s.e.m. of 5–10 independent experiments. *p<0.05, ***p<0.001 compared to untreated MRC-5 fibroblasts (two-tailed student's t-test for paired observations). (TIF) [file pone.0025450.s003.tif]
