# Supplementary material for: Activation of WNT / β-Catenin Signaling in Pulmonary Fibroblasts by TGF-β1 Is Increased in Chronic Obstructive Pulmonary Disease
Source: PLoS One. 2011 Sep 30;6(9):e25450. doi: 10.1371/journal.pone.0025450 (PMC3184127; doi:10.1371/journal.pone.0025450)
Supplement: Table S1 — Primers used for determination of WNT ligands by qRT-PCR analysis. (DOCX) [file pone.0025450.s004.docx]

**Table S1: Primers used for determination of WNT ligands by qRT-PCR analysis**

| **WNT protein family** | **NCBI accession number** | **Primer sequence** | | | |
| --- | --- | --- | --- | --- | --- |
| WNT-1 | [NM_005430](http://www.ncbi.nlm.nih.gov/entrez/viewer.fcgi?db=nucleotide&val=16936523) | Forward | 5' | acc caa tcc ctc tcc act ct | 3' |
|  |  | Reverse | 5' | gat tca agg aaa agc cac ca | 3' |
| WNT-2 | [NM_003391](http://www.ncbi.nlm.nih.gov/entrez/viewer.fcgi?db=nucleotide&val=195230749) | Forward | 5' | caa gaa cgc tga ctg gac aa | 3' |
|  |  | Reverse | 5' | tga ctg cag aac acc agg ag | 3' |
| WNT-2B | [NM_024494](http://www.ncbi.nlm.nih.gov/entrez/viewer.fcgi?db=nucleotide&val=13518020) | Forward | 5' | att tcc cgc tct gga gat tt | 3' |
|  |  | Reverse | 5' | aag ctg gtg caa agg aaa ga | 3' |
| WNT-3 | [NM_030753](http://www.ncbi.nlm.nih.gov/entrez/viewer.fcgi?db=nucleotide&val=21536426) | Forward | 5' | tgt gag gtg aag acc tgc tg | 3' |
|  |  | Reverse | 5' | aaa gtt ggg gga gtt ctc gt | 3' |
| WNT-3A | [NM_033131](http://www.ncbi.nlm.nih.gov/entrez/viewer.fcgi?db=nucleotide&val=17017978) | Forward | 5' | cca cac cgt cag gta ctc ct | 3' |
|  |  | Reverse | 5' | tgt agc tgg atg gag tgc ag | 3' |
| WNT-4 | [NM_030761](http://www.ncbi.nlm.nih.gov/entrez/viewer.fcgi?db=nucleotide&val=156630997) | Forward | 5' | cag gca aga aga ggg agatg | 3' |
|  |  | Reverse | 5' | ccg tgt gtg tgt gtg tgt gt | 3' |
| WNT-5A | [NM_003392](http://www.ncbi.nlm.nih.gov/entrez/viewer.fcgi?db=nucleotide&val=40806204) | Forward | 5' | ggg tgg gaa cca aga aaa at | 3' |
|  |  | Reverse | 5' | tgg aac cta ccc atc cca ta | 3' |
| WNT-5B | [NM_030775](http://www.ncbi.nlm.nih.gov/entrez/viewer.fcgi?db=nucleotide&val=17402918) | Forward | 5' | acg ctg gag atc tct gag ga | 3' |
|  |  | Reverse | 5' | cga ggt tga agc tga gtt cc | 3' |
| WNT-6 | [NM_006522](http://www.ncbi.nlm.nih.gov/entrez/viewer.fcgi?db=nucleotide&val=53729353) | Forward | 5' | gtc acg cag gcc tgt tct at | 3' |
|  |  | Reverse | 5' | cgt cca taa aga gcc tcg ac | 3' |
| WNT-7A | [NM_004625](http://www.ncbi.nlm.nih.gov/entrez/viewer.fcgi?db=nucleotide&val=34328912) | Forward | 5' | ccc acc ttc ctg aag atc aa | 3' |
|  |  | Reverse | 5' | aca gca cat gag gtc aca gc | 3' |
| WNT-7B | [NM_058238](http://www.ncbi.nlm.nih.gov/entrez/viewer.fcgi?db=nucleotide&val=17505192) | Forward | 5' | gcc tgc agg tcc tag aag tg | 3' |
|  |  | Reverse | 5' | ctc cca aag tgc tgg gat ta | 3' |
| WNT-8A | [NM_058244](http://www.ncbi.nlm.nih.gov/entrez/viewer.fcgi?db=nucleotide&val=89111138) | Forward | 5' | tgc aag ttc cag ttt gct tg | 3' |
|  |  | Reverse | 5' | atc ctt tcc cca aat tcc ac | 3' |
| WNT-8B | [NM_003393](http://www.ncbi.nlm.nih.gov/entrez/viewer.fcgi?db=nucleotide&val=110735436) | Forward | 5' | cca tga acc tgc aca aca ac | 3' |
|  |  | Reverse | 5' | tga gtg ctg cgt ggt act tc | 3' |
| WNT-9A | [NM_003395](http://www.ncbi.nlm.nih.gov/entrez/viewer.fcgi?db=nucleotide&val=15082260) | Forward | 5' | tga gaa gaa ctg cga gag ca | 3' |
|  |  | Reverse | 5' | ctg tgt gca atg cct gta cc | 3' |
| WNT-9B | [NM_003396](http://www.ncbi.nlm.nih.gov/entrez/viewer.fcgi?db=nucleotide&val=17017975) | Forward | 5' | gag gac tca ccc agc ttc tg | 3' |
|  |  | Reverse | 5' | tag gcc tag tgc ttg cag gt | 3' |
| WNT-10A | [AK315081](http://www.ncbi.nlm.nih.gov/entrez/viewer.fcgi?db=nucleotide&val=164693883) | Forward | 5' | aag ctg cac cgc tta caa ct | 3' |
|  |  | Reverse | 5' | att ctc gcg tgg atg tct ct | 3' |
| WNT-10B | [NM_003394](http://www.ncbi.nlm.nih.gov/entrez/viewer.fcgi?db=nucleotide&val=16936521) | Forward | 5' | aat gcg aat cca caa caa ca | 3' |
|  |  | Reverse | 5' | ggg tct cgc tca cag aag tc | 3' |
| WNT-11 | [NM_004626](http://www.ncbi.nlm.nih.gov/entrez/viewer.fcgi?db=nucleotide&val=17017973) | Forward | 5' | act ctg ctc aag gac cct ca | 3' |
|  |  | Reverse | 5' | gct tcc aag tga agg caa ag | 3' |
| WNT-16 | [NM_016087](http://www.ncbi.nlm.nih.gov/entrez/viewer.fcgi?db=nucleotide&val=17402913) | Forward | 5' | gct cct gtg ctg tga aaa ca | 3' |
|  |  | Reverse | 5' | acc ctc tga tgt acg gtt gc | 3' |
