# Supplementary material for: Activation of WNT / β-Catenin Signaling in Pulmonary Fibroblasts by TGF-β1 Is Increased in Chronic Obstructive Pulmonary Disease
Source: PLoS One. 2011 Sep 30;6(9):e25450. doi: 10.1371/journal.pone.0025450 (PMC3184127; doi:10.1371/journal.pone.0025450)
Supplement: Table S2 — Primers used for determination of the dishevelled protein family by qRT-PCR analysis. (DOCX) [file pone.0025450.s005.docx]

**Table S2:** **Primers used for determination of the dishevelled protein family by qRT-PCR analysis**

| **Dishevelled protein family** | | |  | **Primer sequence** |  |
| --- | --- | --- | --- | --- | --- |
| DVL1 | [NM_004421](http://www.ncbi.nlm.nih.gov/entrez/viewer.fcgi?db=nucleotide&val=32479520) | Forward | 5' | acc ctg aac ctc aac agt gg | 3' |
|  |  | Reverse | 5' | ccc ttc act ctg ctg act cc | 3' |
| DVL2 | [NM_004422](http://www.ncbi.nlm.nih.gov/entrez/viewer.fcgi?db=nucleotide&val=48762939) | Forward | 5' | ccg tca tgt gct tgc tct ta | 3' |
|  |  | Reverse | 5' | tgg agg agg agg tca cat tc | 3' |
| DVL3 | [NM_004423](http://www.ncbi.nlm.nih.gov/entrez/viewer.fcgi?db=nucleotide&val=41406096) | Forward | 5' | gag gct gag gca caa gaa tc | 3' |
|  |  | Reverse | 5' | gca ggc aag att gag tca ca | 3' |
