# Supplementary material for: Activation of WNT / β-Catenin Signaling in Pulmonary Fibroblasts by TGF-β1 Is Increased in Chronic Obstructive Pulmonary Disease
Source: PLoS One. 2011 Sep 30;6(9):e25450. doi: 10.1371/journal.pone.0025450 (PMC3184127; doi:10.1371/journal.pone.0025450)
Supplement: Table S3 — Primers used for determination of FZD receptors by qRT-PCR analysis. (DOCX) [file pone.0025450.s006.docx]

**Table S3: Primers used for determination of FZD receptors by qRT-PCR analysis**

| **Frizzled protein family** | |  |  | **Primer sequence** |  |
| --- | --- | --- | --- | --- | --- |
| FZD_1_ | [NM_003505](http://www.ncbi.nlm.nih.gov/entrez/viewer.fcgi?db=nucleotide&val=4503824) | Forward | 5' | tcg act tcc tga agc tgg at | 3' |
|  |  | Reverse | 5' | aag gtg gga gaa ggg agt gt | 3' |
| FZD_2_ | [NM_001466](http://www.ncbi.nlm.nih.gov/entrez/viewer.fcgi?db=nucleotide&val=5922012) | Forward | 5' | ccc gact tcac ggt cta cat | 3' |
|  |  | Reverse | 5' | ctg ttg gtg agg cga gtg ta | 3' |
| FZD_3_ | [NM_017412](http://www.ncbi.nlm.nih.gov/entrez/viewer.fcgi?db=nucleotide&val=22035685) | Forward | 5' | tct ctt tgg ccc ttg act g | 3' |
|  |  | Reverse | 5' | aca aag aaa agg ccg gaa at | 3' |
| FZD_4_ | [NM_012193](http://www.ncbi.nlm.nih.gov/entrez/viewer.fcgi?db=nucleotide&val=22547160) | Forward | 5' | cca gga ttc ctt cca agt ca | 3' |
|  |  | Reverse | 5' | cca tgt cct tgt ggc cta ct | 3' |
| FZD_5_ | [NM_003468](http://www.ncbi.nlm.nih.gov/entrez/viewer.fcgi?db=nucleotide&val=111160871) | Forward | 5' | agc taa aat ggc cag agc aa | 3' |
|  |  | Reverse | 5' | aat tcc ccc tgg gaa cta tg | 3' |
| FZD_6_ | [NM_003506](http://www.ncbi.nlm.nih.gov/entrez/viewer.fcgi?db=nucleotide&val=34734078) | Forward | 5' | ttg ttg gca tct ctg ctg tc | 3' |
|  |  | Reverse | 5' | cca tgg att tgg aaa tga cc | 3' |
| FZD_7_ | [NM_003507](http://www.ncbi.nlm.nih.gov/entrez/viewer.fcgi?db=nucleotide&val=4503832) | Forward | 5' | cga cgc tct tta ccg ttc tc | 3' |
|  |  | Reverse | 5' | gcc atg ccg aag aag tag ag | 3' |
| FZD_8_ | [NM_031866](http://www.ncbi.nlm.nih.gov/entrez/viewer.fcgi?db=nucleotide&val=13994189) | Forward | 5' | gac act tga tgg gct gag gt | 3' |
|  |  | Reverse | 5' | caa atc tcg ggt tct gga aa | 3' |
| FZD_9_ | [NM_003508](http://www.ncbi.nlm.nih.gov/entrez/viewer.fcgi?db=nucleotide&val=62865872) | Forward | 5' | aga cca tcg tca tcc tga cc | 3' |
|  |  | Reverse | 5' | cca tga gct tct cca gct tc | 3' |
| FZD_10_ | [NM_007197](http://www.ncbi.nlm.nih.gov/entrez/viewer.fcgi?db=nucleotide&val=22035684) | Forward | 5' | cct cca aga ctc tgc agt cc | 3' |
|  |  | Reverse | 5' | gac tgg gca ggg atc tca ta | 3' |
