# Supplementary material for: Activation of WNT / β-Catenin Signaling in Pulmonary Fibroblasts by TGF-β1 Is Increased in Chronic Obstructive Pulmonary Disease
Source: PLoS One. 2011 Sep 30;6(9):e25450. doi: 10.1371/journal.pone.0025450 (PMC3184127; doi:10.1371/journal.pone.0025450)
Supplement: Table S4 — Primers used for determination of LRP-coreceptors, WNT target genes and housekeeping genes by qRT-PCR analysis. (DOCX) [file pone.0025450.s007.docx]

**Table S4: Primers used for determination of LRP-coreceptors, WNT target genes and housekeeping genes by qRT-PCR analysis**

| LRP-coreceptors and  WNT-target genes | |  |  | Primer sequence |  |
| --- | --- | --- | --- | --- | --- |
| LRP5 | [NM_002335](http://www.ncbi.nlm.nih.gov/entrez/viewer.fcgi?db=nucleotide&val=119709831) | Forward | 5' | gca gga ggg gaa gct cta ct | 3' |
|  |  | Reverse | 5' | gta gat gaa gtc ccc cag ca | 3' |
| LRP6 | [NM_002336](http://www.ncbi.nlm.nih.gov/entrez/viewer.fcgi?db=nucleotide&val=148727287) | Forward | 5' | ccc atg cac ctg gtt cta ct | 3' |
|  |  | Reverse | 5' | cca agc cac agg gat aca gt | 3' |
| β-Catenin | [NM_00](http://www.ncbi.nlm.nih.gov/entrez/viewer.fcgi?db=nucleotide&val=148727287)1904 | Forward | 5' | CCC ACT AAT GTC CAG CGT TT | 3' |
|  |  | Reverse | 5' | AAT CCA CTG GTG AAC CAA GC | 3' |
| Collagen1α1 | NM_000088 | Forward | 5' | AGC CAG CAG ATC GAG AAC AT | 3' |
|  |  | Reverse | 5' | TCT TGT CCT TGG GGT TCT TG | 3' |
| α-sm-actin | NM_001141945 | Forward | 5' | GAC CCT GAA GTA CCC GAT AGA AC | 3' |
|  |  | Reverse | 5' | GGG CAA CAC GAA GCT CAT TG | 3' |
| Fibronectin | NM_212482 | Forward | 5' | TCG AGG AGG AAA TTC CAA TG | 3' |
|  |  | Reverse | 5' | ACA CAC GTG CAC CTC AT CAT | 3' |
| MMP-2 | NM_004530.4 | Forward | 5' | ACA TCA AGG GCA TTC AGG AG | 3' |
|  |  | Reverse | 5' | GCC TCG TAT ACC GCA TCA AT | 3' |
| VEGF | NM_001171623.1 | Forward | 5' | CTA CCT CCA CCA TGC CAA GT | 3' |
|  |  | Reverse | 5' | TGG TGA TGT TGG ACT CCT CA | 3' |
| IL-8 | NM_000584.3 | Forward | 5' | TAG CAA AAT TGA GGC CAA GG | 3' |
|  |  | Reverse | 5' | AAA CCA AGG CAC AGT GGA AC | 3' |
| DKK-1 | NM_12242.2 | Forward | 5' | ATG GAA CTC CCC TGT GAT TG | 3' |
|  |  | Reverse | 5' | AAT AGG CAG TGC AGC ACC TT | 3' |
| AXIN-2 | NM_0044655.3 | Forward | 5' | CCT GCC ACC AAG ACC TAC AT | 3' |
|  |  | Reverse | 5' | CTT CAT TCA AGG TGG GGA GA | 3' |
| PAI-1 | NG_013213.1 | Forward | 5' | CGC CAG AGC AGG ACG AA | 3' |
|  |  | Reverse | 5' | GGA CAC ATC TGC ATC CTG AAG TT | 3' |
| 18S rRNA | NR_003286.2 | Forward | 5' | CGC CGC TAG AGG TGA AAT TC | 3' |
|  |  | Reverse | 5' | TTG GCA AAT GCT TTC GCT C | 3' |
| B2M | NM_004048 | Forward | 5’ | TGC TGT CTC CAT GTT TGA TGT ATC T | 3’ |
|  |  | Reverse | 5’ | TCT CTG CTC CCC ACC TCT AAG T | 3’ |
| YWHAZ | NM_003406 | Forward | 5' | ACT TTT GGT ACA TTG TGG CTT CAA | 3' |
|  |  | Reverse | 5' | CCG CCA GGA CAA ACC AGT AT | 3' |
